# Supplementary figures and images for: Oligodendrocyte-derived IL-33 functions as a microglial survival factor during neuroinvasive flavivirus infection
Source: PLoS Pathog. 2023 Nov 20;19(11):e1011350. doi: 10.1371/journal.ppat.1011350 (PMC10695366; doi:10.1371/journal.ppat.1011350)

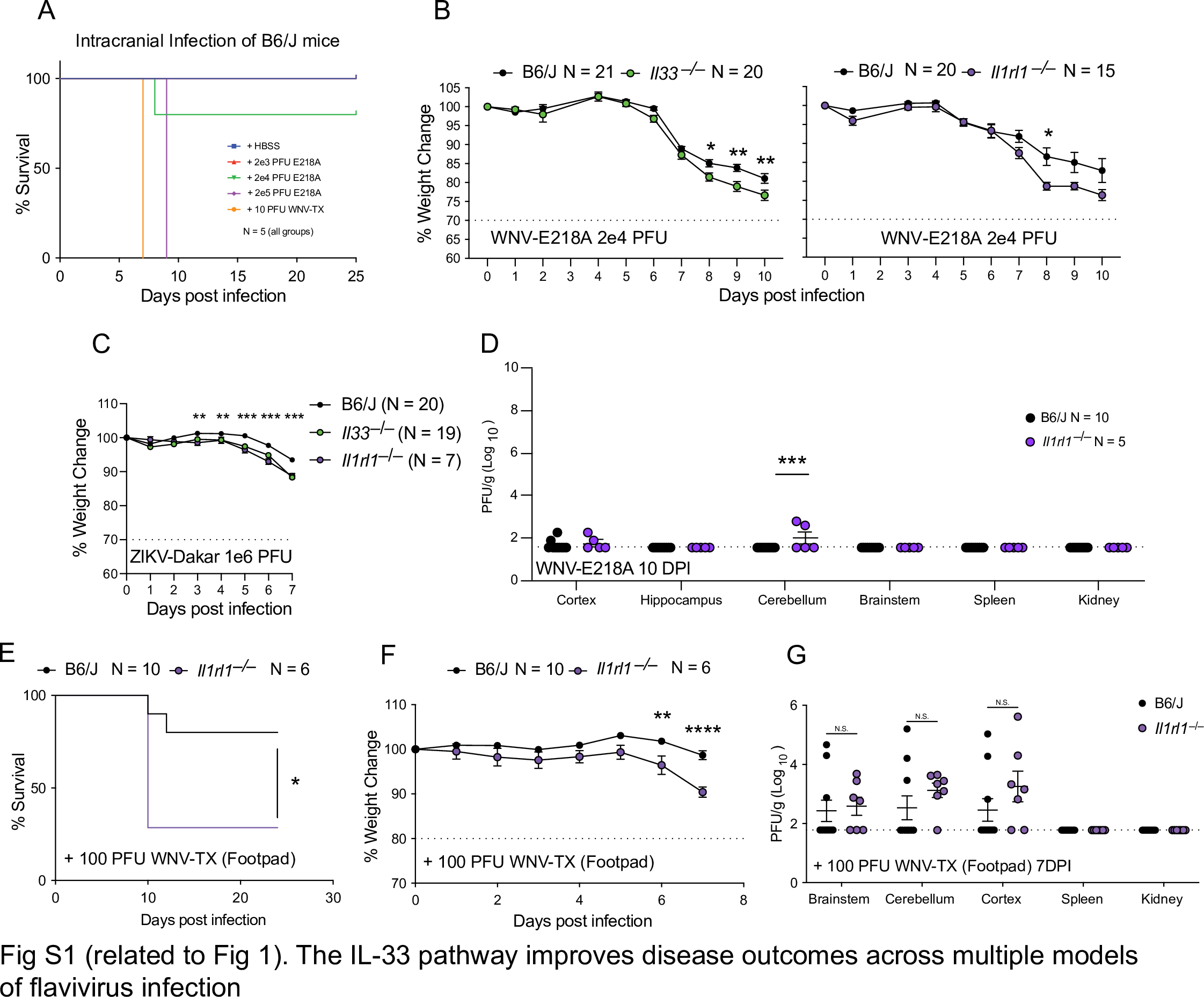

Supplement: S1 Fig — (A) Survival analysis of adult B6/J mice exposed to increasing doses of intracranial WNV-E218A and WNV-Tx. (B) Weight loss of Il33–/–, Il1rl1–/–, and B6/J mice following 2e4 PFU WNV-E218A intracranial infection. (C) Weight loss of Il33–/–, Il1rl1–/–, and B6/J mice following 1e6 PFU ZIKV-Dakar intracranial infection. (D) WNV Viral titers from indicated CNS components and tissues of mice 10 days post-infection via plaque assay on BHK cells. (E) Survival analysis of Il1rl1–/–and B6/J mice following footpad infection with 100 PFU WNV-Tx. (F) Weight loss of Il1rl1–/–and B6/J mice following footpad infection with 100 PFU WNV-Tx. (G) WNV-Tx Viral titers from indicated CNS components and tissues of mice 7 days post-foot pad infection via plaque assay on BHK cells. Data are representative of 3 pooled independent experiments (error bars, SEM). * p < 0.05, ** p < 0.01, *** p < 0.001 (A, D) (Gehan-Breslow-Wilcoxon test) (B, C, E) (2-way ANOVA with Holm-Sidak Multiple comparisons). (TIF) [file ppat.1011350.s001.tif]

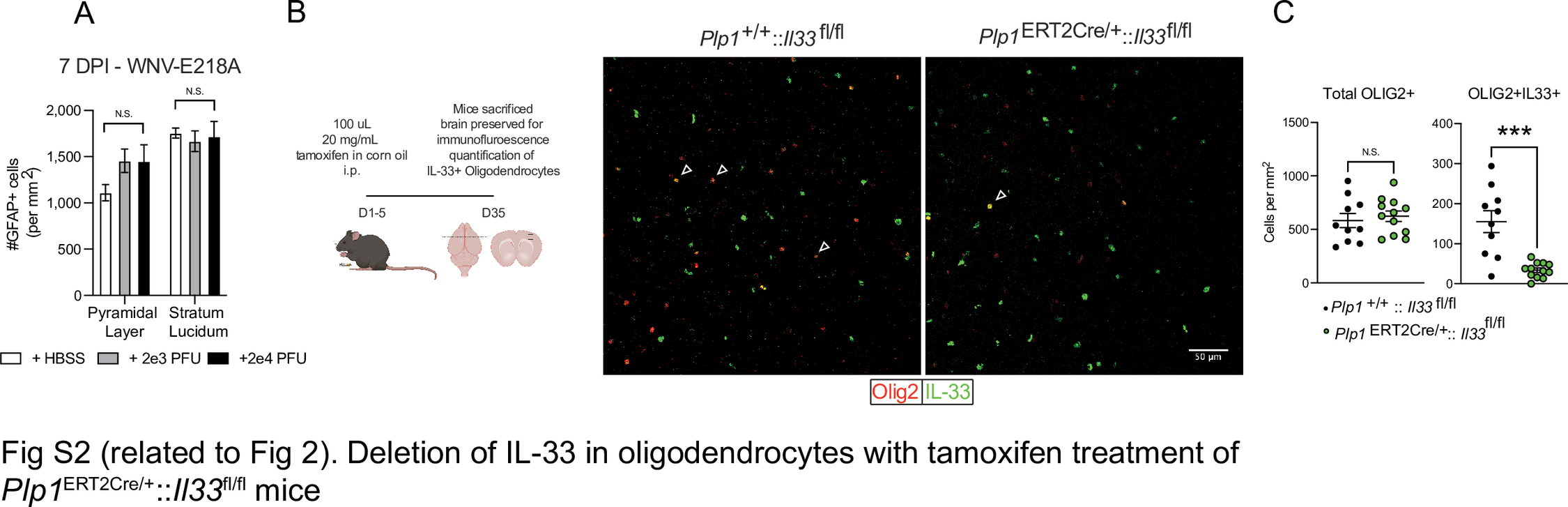

Supplement: S2 Fig — (A) GFAP+ cell number by immunohistochemistry following intracranial infection with increasing doses of WNV-E218A. (B) Scheme for tamoxifen injections to induce IL-33 excision (created using Biorender.com and exported via an academic subscription belonging to AO) and representative images of IL-33 loss in cortical oligodendrocytes following tamoxifen treatment (20x magnification, scale bars, 50 microns). Arrowheads Indicate Olig2+IL-33+ cells. (C) Quantification of total cortical oligodendrocyte number and IL-33+ oligodendrocytes following tamoxifen injection. Data are representative of 3 pooled independent experiments (error bars, SEM). *** p < 0.001. (C) two-tailed student’s T test. (TIF) [file ppat.1011350.s002.tif]

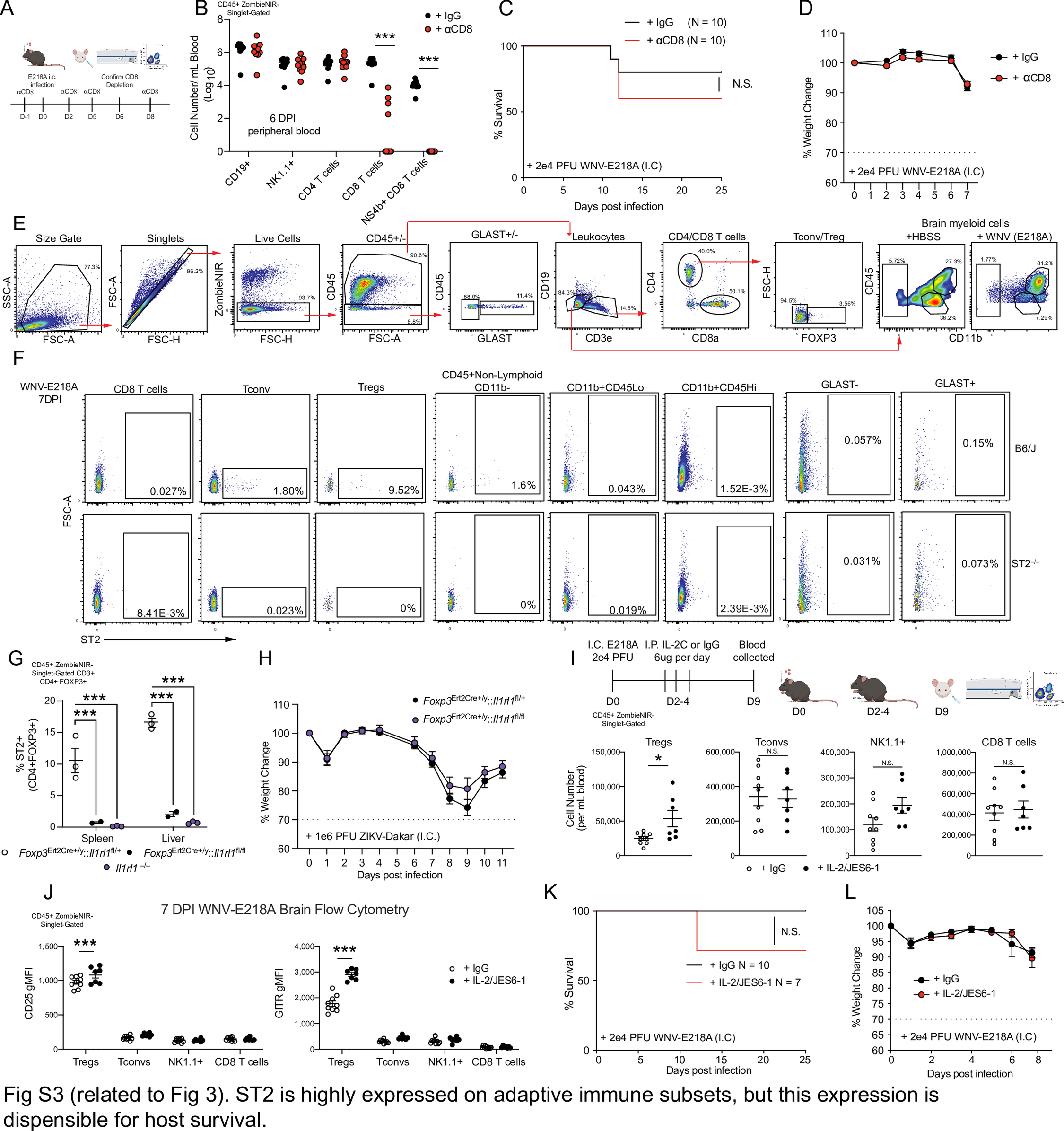

Supplement: S3 Fig — (A) Scheme for CD8 T cell depletion during intracranial WNV-E218A infection, created using Biorender.com and exported via an academic subscription belonging to AO. (B) Peripheral blood counts 6 DPI with WNV-E218A to confirm CD8 depletion. (C) Survival analysis of mice with CD8 T cell depletion and WNV-E218A intracranial infection. (D) Weight change in mice with CD8 T cell depletion and control IgG injection following WNV-E218A intracranial infection. (E) Representative FACs plots and gating strategy for indicated cell types from brain homogenate 7 days post intracranial WNV-E218A infection. (F) Representative FACs plots of ST2 expression on indicated cell types from B6/J and Il1rl1–/–brain homogenate 7 days post intracranial WNV-E218A infection. (G) Quantification of ST2 excision on splenic and liver Tregs of indicated genotype one-month post tamoxifen treatment. (H) Weight change in mice with Treg ST2 deletion following intracranial infection with ZIKV-Dakar. (I) Scheme and cell number from peripheral blood of indicated immune cells following IL-2 complex treatment and WNV-E218A infection at 9 DPI. (J) gMFI of CD25 and GITR on peripheral blood Tregs at 9 DPI with WNV-E218A and IL-2 complex treatment. (K) Survival analysis of mice with control or IL-2 complex treatment following intracranial infection with WNV-E218A. (L) Weight change in mice with control or IL-2 complex treatment after intracranial infection with WNV-E218A. Data are representative of 2 pooled independent experiments (error bars, SEM). * p < 0.05, ** p < 0.01, *** p < 0.001. (B, D, G, J, H, L) (2-way ANOVA with Holm-Sidak Multiple comparisons) (C, K) (Gehan-Breslow-Wilcoxon test) (I) (Student’s two-tailed t Test). (TIF) [file ppat.1011350.s003.tif]

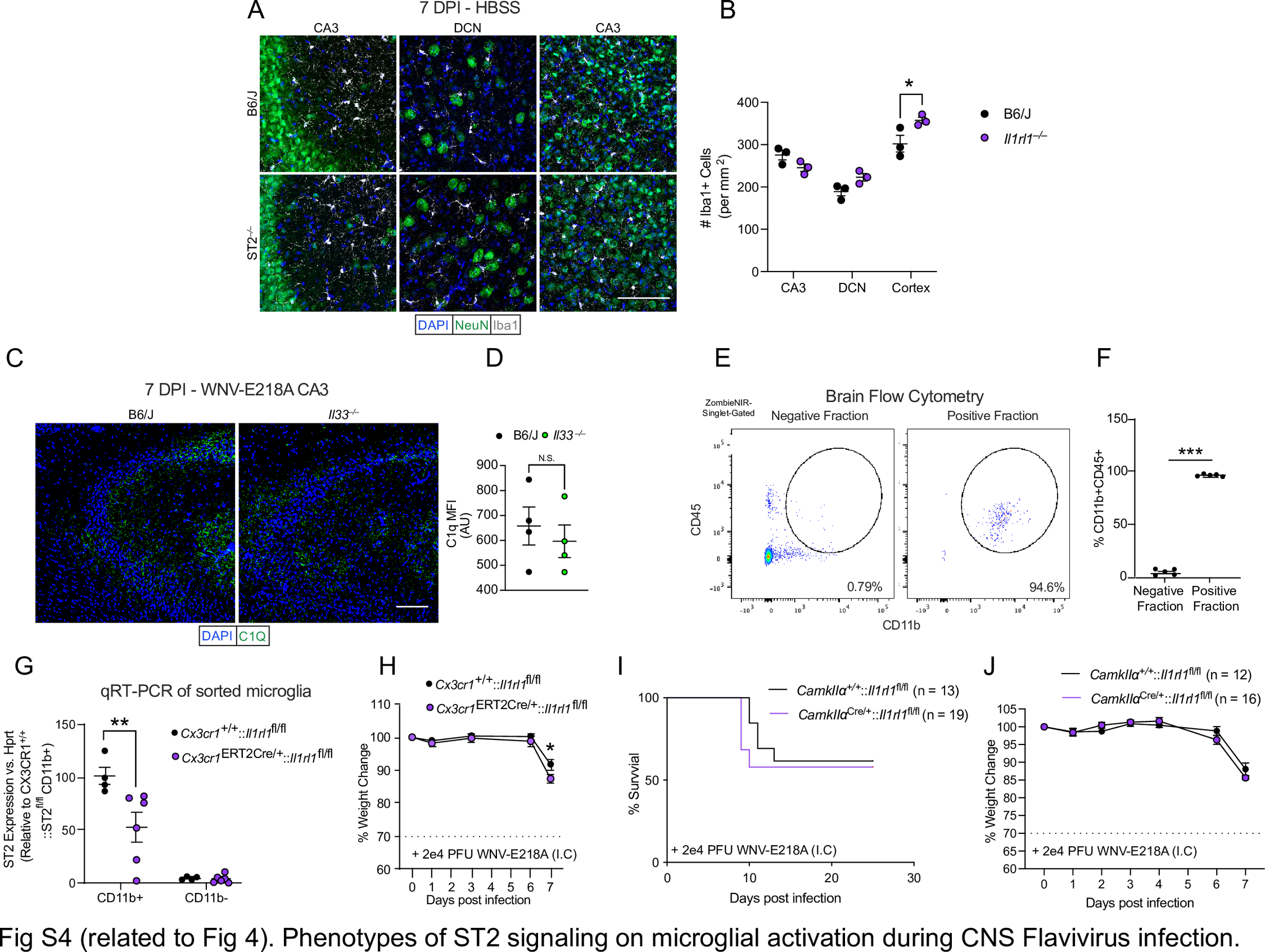

Supplement: S4 Fig — (A) Representative immunofluorescent images of Iba1+ brain macrophages in B6/J and Il1rl1–/–mice 7 days post vehicle injection (20x magnification, scale bars, 100 microns). (B) Quantification of Iba1+ cell number in vehicle injected mice of indicated brain regions. (C) Representative immunofluorescent images of C1q deposition in hippocampal CA3 of B6/J and Il33–/–mice 7 days post-infection (20x magnification, scale bars, 100 microns). (D) Quantification of C1q gMFI by immunofluorescence in hippocampal CA3 of B6/J and Il33–/–mice 7 days post-infection. (E) Representative FACs plots of microglia from CX3CR1ERT2Cre/+:: Il1rl1fl/fl 30 days post tamoxifen treatment and following CD11b enrichment by MACs. (F) Quantification of MACs enrichment efficiency. (G) Quantification of Il1rl1 RNA by qRT-PCR of sorted microglia 30 days post tamoxifen treatment and following CD11b enrichment by MACs. (H) Weight change in CX3CR1ERT2Cre/+:: Il1rl1fl/fl mice following intracranial infection of WNV-E218A. (I) Survival analysis of mice with neuronal Il1rl1 deletion following intracranial infection with WNV-E218A. (J) Weight change of mice with neuronal Il1rl1 deletion following intracranial infection with WNV-E218A. Data are representative of 2 (A-F) independent and 2 (G-N) pooled independent experiments (error bars, SEM). * p < 0.05, ** p < 0.01, *** p < 0.001. (B, F, H, L) (two-tailed T test) (D, I, J, N) (Two-way ANOVA with Holm-Sidak multiple comparisons) (M) (Gehan-Breslow-Wilcoxon test). (TIF) [file ppat.1011350.s004.tif]

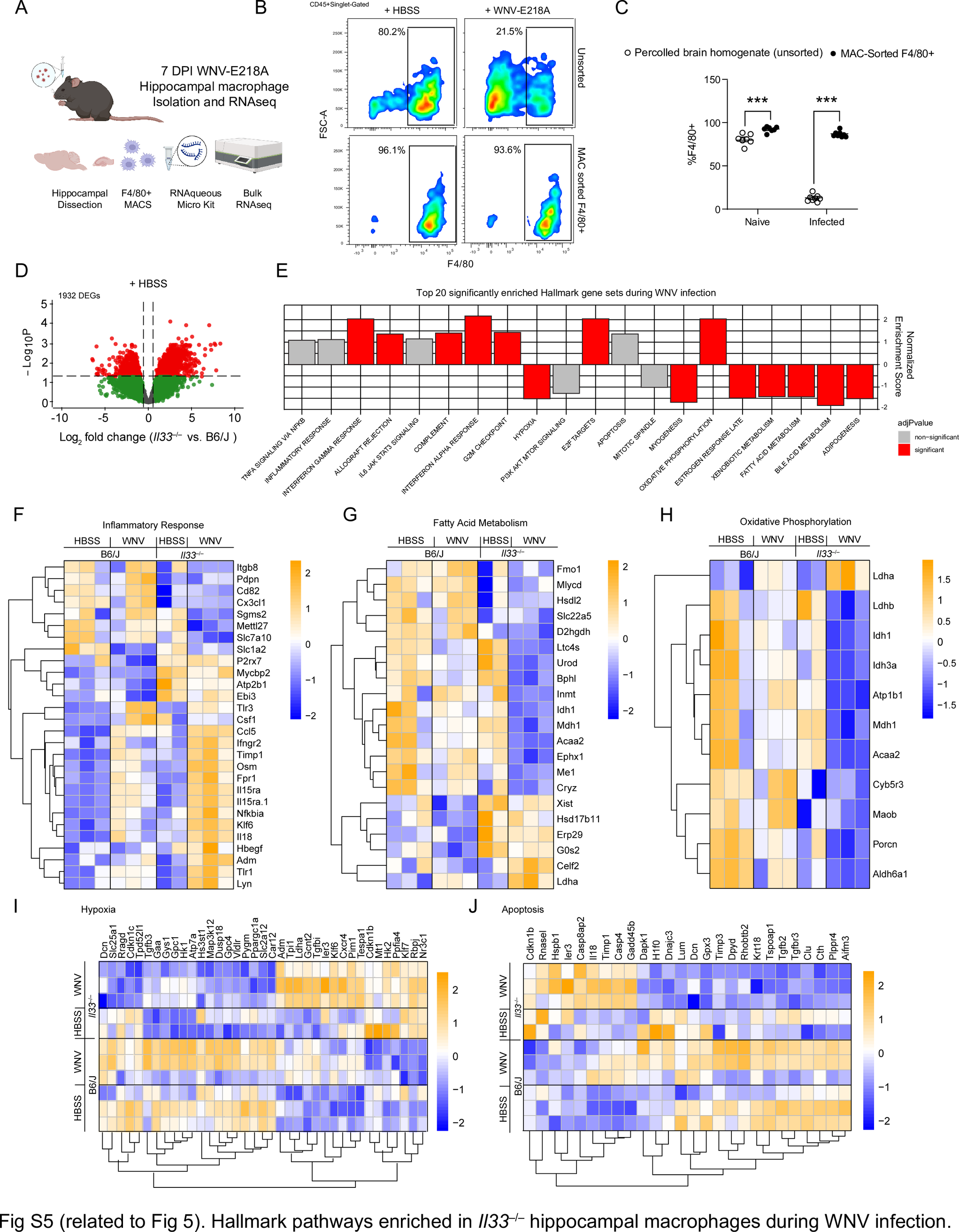

Supplement: S5 Fig — (A) Scheme of hippocampal macrophage isolation and RNAsequencing following intracranial WNV-E218A infection of B6/J and Il33–/–mice, created using Biorender.com and exported via an academic subscription belonging to AO. (B) Representative FACS plots of MACs-sorted samples. (C) Quantification of sample purity by FACS post-MAC-sort. (D) Volcano plot showing enriched genes in Il33–/–hippocampal macrophages relative to B6/J with vehicle injection. (E) Top 20 significantly enriched Hallmark gene sets by GSEA of Il33–/–and B6/J hippocampal macrophages post-infection in vehicle-injected samples. (F) Heatmap of Inflammatory Response pathway genes in indicated samples. (G) Heatmap of Fatty Acid Metabolism pathway genes in indicated samples. (H) Heatmap of Oxidative Phosphorylation pathway genes in indicated samples. (I) Heatmap of Hypoxia pathway genes in indicated samples. (J) Heatmap of Apoptosis pathway genes in indicated samples. *** p < 0.001 Two-way ANOVA with Holm-Sidak multiple comparisons. (TIF) [file ppat.1011350.s005.tif]

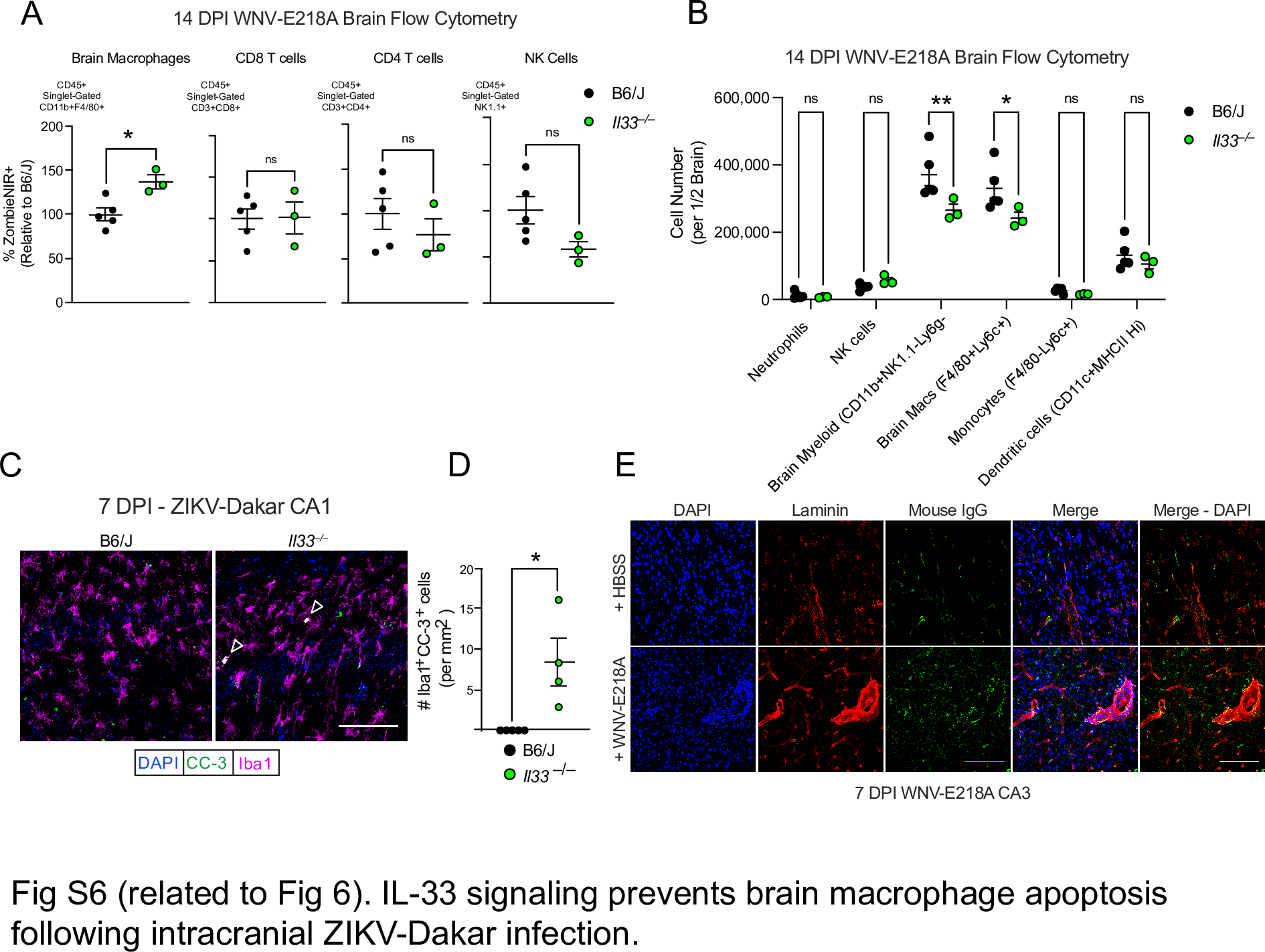

Supplement: S6 Fig — (A) %ZombieNIR+ dying cell populations of indicated type by FACs analysis of brain homogenates 14 days post-infection with WNV-E218A in B6/J and Il33–/–mice. (B) Cell number of indicated innate immune cell types per ½ brain by FACs 14 days post intracranial infection with WNV-E218A. (C) Representative images of CC3+ hippocampal CA1 brain macrophages in B6/J and Il33–/–mice 7 days post-infection with WNV-E218A (20x magnification, scale bars, 100 microns). (D) Quantification of CC3+ hippocampal CA1 brain macrophages in B6/J and Il33–/–mice. (E) Representative images of perivascular IgG staining in hippocampal CA3 in 7 days post-infection WNV-E218A and HBSS-injected brains from B6/J mice (20x magnification, scale bars, 100 microns). (TIF) [file ppat.1011350.s006.tif]
