# Supplementary material for: Oligodendrocyte-derived IL-33 functions as a microglial survival factor during neuroinvasive flavivirus infection
Source: PLoS Pathog. 2023 Nov 20;19(11):e1011350. doi: 10.1371/journal.ppat.1011350 (PMC10695366; doi:10.1371/journal.ppat.1011350)
Supplement: Attachment — Submitted filename: Response to Reviewers.docx [file ppat.1011350.s007.docx]

We would like to thank the reviewers and the editors for their comments; addressing these concerns has strengthened our manuscript. Below, we provide a point-by-point response to each reviewer’s comments, provided *in blue italics*.

**Reviewer #1**:

Figure 1. There is a substantial difference in the survival curve of WT versus IL-33- or IL1rl1-deficient mice after infection with WNV-E218A. The KO mice appear to die earlier, suggesting a loss of virologic control by innate immune mechanisms, perhaps in the periphery. The authors should include cervical draining LNs in their virologic analyses to address this possibility.

*We now include titers from cervical draining lymph nodes at both early and late timepoints after infection (Figure 3A). These assays reveal no infectious WNV in this tissue at either timepoint, suggesting that IC infection confines viral dissemination to the CNS.*

There is also a substantial difference in the survival curves observed comparing ZIKV-Dakar infected IL-33- versus IL1rl1-deficient mice. Is this difference significant? These data suggest an alternative IL-33 receptor is involved in survival from ZIKV-Dakar infection.

*These differences have been quantified and included in the revised text. Notably, IL-33 has also been described to have a role in the nucleus independent of its alarmin function. We cannot formally exclude the possibility that this function plays a role in survival independent of ST2, or that another receptor recognizes IL-33 in ST2 (Il1rl1) knockout mice.*

Also, the trend towards decreased viral loads within the brainstem of IL1rl1-deficient mice infected with ZIKV-Dakar should be further evaluated as this is consistent with the improved survival curve for this genotype compared with IL-33 KO mice. Importantly, a kinetic analysis of viral loads over time, in both rostral and caudal CNS, is required to conclude that loss of IL-33 or its receptors does not impact viral clearance. The difference in viral clearance within the CB by 10 dpi between WT and IL1rl1-deficient mice supports the need for this additional analysis.

*We now include viral load analysis at day 4 and day 14 post-infection across several brain regions as well as the periphery (Fig. 1E-F). These additional analyses do not reveal consistent differences in viral load in IL-33-deficient animals at either timepoint, consistent with the suggestion that IL-33 promotes survival independent of control of WNV proliferation.*

Figure 2. Olig2 is expressed by oligodendrocyte precursor cells and by multipotential polydendrocytes, as is NG2, which could help identify these cells. In the HIP, about 76% of Olig2+ cells have NG2 co-expression, which is the highest among selected regions ([doi.org/10.1186/s13041-021-00747-0](http://doi.org/10.1186/s13041-021-00747-0)). Plp immunostaining is a better marker of mature oligodendrocytes, which would also show their processes.

*This is a good point, and was also raised by Reviewer 2. We now include immunohistochemistry co-staining of mature oligodendrocytes using the marker MBP, along with IL-33. Analogous to our previous findings using the Olig2 marker, this analysis shows loss of IL-33 positive mature oligodendrocytes upon WNV infection. These data appear in Figs. 2D and 2E.*

Finally, the robust loss of survival phenotype observed in WNV-infected mice with IL-33 deletion in oligodendrocytes is striking. It is unclear why this result is not follow-up at all.

*We now include whole-brain and peripheral organ titers from mice with conditional loss of IL-33 in oligodendrocytes. These data largely mirror similar experiments performed using whole-animal knockout of IL-33; the goal of this experiment is to demonstrate that loss of IL-33 from oligodendrocytes is sufficient to explain the phenotype observed in whole-animal knockouts. We therefore consider the remainder of the manuscript to be a further exploration of this finding.*

Figure 4. At 7 dpi, monocytes heavily infiltrate the WNV-infected CNS and microglia become activated. As both express Iba1, this molecule cannot distinguish between these cells. Microglial specific markers should be used to distinguish effects on infiltrating versus resident myeloid cells. The authors cannot conclude effects specifically for microglia without these tools.

*This is a good point. We now include co-staining with Iba1 and the microglial marker TMEM119 (Fig. 4A). This analysis reveals many Iba1+/TMEM119- cells, consistent with either loss of TMEM119 due to immune activation or a significant infiltration by peripheral monocytes, though it does not alter our conclusions regarding roles for IL-33 in myeloid cell activation in the brain. We have carefully edited the manuscript to refer to “microglia” only when we can confirm the identity of these cells, and to “brain macrophages” when we cannot.*

The correlations between IL-33 and Iba1 is poor. The data evaluating Iba1 at 10 dpi is interesting, but it is unclear whether there is loss of the macrophages or activated microglia. Microglial markers are needed to determine this. Similarly, the RNAseq experiment cannot distinguish between these two cell types. Finally, as CX3CR1 is expressed by memory T cells during viral clearance (10.1038/ncomms9306), the CreLox experiment does not specifically target microglia.

*Please see the response above regarding additional staining of TMEM119 to validate microglial specificity.*

*Regarding the microglial specificity of CX3CR1-Cre, please note that we used a well-validated strategy to restrict deletion to microglia (see e.g. PMID 24360280). Inducible CX3CR1-CreERT2 as activated via tamoxifen administration, deleting ST2 in all CX3CR1-expressing cells. Mice were then rested for one month; previous studies, validated by our own findings, indicate that this time allows peripheral CX3CR1 expressing cells to turn over and be replaced by wild-type cells, while self-renewing microglial populations remain ablated for the target gene. A citation of previous studies validating this approach, and a clearer explanation of this strategy has been added to the manuscript.*

*In addition, please see the answer to Reviewer 2’s critique below. We now include additional analysis, suggested by our RNAseq dataset, of p38 pathway activation in CD68/Iba1+ cells. This analysis more clearly demonstrates activation defects in microglia following WNV infection in IL-33 deficient animals.*

Figure 5. In this figure the authors isolated myeloid cells for RNAseq. However, it is unclear whether the alterations in gene expression are due to effects on macrophages or microglia, or even on differential recruitment of myeloid cells. The authors need to address this issue throughout the manuscript.

*We agree that our experimental procedure for RNAseq analysis does not allow direct differentiation between microglia and macrophages. This debate is an old and well-worn one in the neuroinflammation field, and we do not claim or expect to settle it here. To avoid confusion, we use the term “brain macrophage” to indicate that the cells analyzed are myeloid in origin, but may be microglia or engrafted peripheral monocyte/macrophages.*

Figure 6. The IgG detection is not consistent with BBB disruption - the IgG would be localized strongly to vessels and fan out from them into the parenchyma. The increase in GFAP is consistent with immune activation of astrocytes, however, why was this not observed in FigS2?

*To clarify this point, we now include co-staining of IgG along with the endothelial marker laminin, 7 days after infection with WNV. This co-staining does show that the IgG deposition we observe co-localizes with vessels, as suggested by the reviewer. These data appear in Fig. S6E.*

*Regarding Figure S2, please note that it depicts GFAP+ cell number rather than intensity of GFAP signal, consistent with the data presented in Figure 6. We have updated the manuscript text to better clarify this point.*

**Reviewer #2**: Importantly, the authors conclude that microglia activation (Iba-1 MFI) and microglia apoptosis are influence by IL-33/ST2. In figure 6A, the number of dead microglia in the tissue prep are increased, but it is not clear (unless I missed it) whether live microglia number decreases in ST2 or IL-33 knockout mice. To this end, Iba-1 MFI is clearly decreased in knockout mice, but is this due to the absence of the cells or the lack of “activation.” The title of Figure 4 conveys the conclusion that microglia activation is reduced in the absence of IL-33 based primarily on Iba-1 intensity. Because this marker is also used to show the presence of cell (including some monocyte-derived cells which appear to increase in ko mice), it is difficult to support a conclusion for “activation” based on Iba-1 alone. Perhaps some “activation” genes from the RNAseq dataset could be helpful for further demonstrating this point. For example, reduced expression of MHC molecules, ccl2, or itgax and higher expression of p2ry12, sall1/3, or fcrls in microglia from knockout mice would greatly support this conclusion.

*This is a good suggestion. To address it, we analyzed our RNAseq dataset, as well as previously published datasets measuring the response of glial cells to IL-33. Previous literature suggested that the p38 pathway was robustly activated downstream of IL-33, and might serve as a better diagnostic marker of IL-33-meidated microglial activation than Iba1 staining. We therefore carried out co-staining analysis of phospho-p38, along with the Iba1 and the phagocytosis / activation marker CD68. This analysis revealed remarkably lower expression of p-p38 and CD68 in IL-33-deficient mice following WNV infection, consistent with defects in microglia/macrophage activation in this setting. These new data appear in Figure 4 B-C.*

**Reviewer #3**-

The authors must consider previous literature reports describing the role of IL-33 signaling in the CNS in the context of viral infections, there are at least two relavent papers (doi: 10.1186/s12974-016-0628-1 DOI: 10.1371/journal.ppat.1006322) describing the participation of ST2 signaling in neuronal infections and these must be considered in the introduction.

*We thank the reviewer for bringing these papers to our attention; they are now duly cited.*

"Since WNV-E218A is an attenuated flavivirus sensitive to the interferon stimulated gene IFIT1, we tested the effects of IL-33 and ST2 deletion with a wild-type isolate of the related flavivirus Zika virus, termed ZIKV-Dakar"I fail to understand the logic behind this, as ZIKV is also known to be sensitive to type I IFN. Therefore, this explanation appears irrelevant. I believe the rationale behind using another neurotropic virus is to verify whether IL-33 signaling in the central nervous system (CNS) is exclusively associated with WNV or if it has a broader role in other CNS viral infections as well.

*This is a fair point, and we have edited the manuscript to reflect this concern.*

Figure 2 - the source of IL-33 is hard to appreciate. These data is not convincing and other experiments should be performed to conclude that IL-33 is being produced mainly by Oligo2+ cells. Would be possible to perform a cytometry analysis, staining the cells for IL-33 and specific markers? Also, is OLIG2 an specific marker for oligodendrocytes or is it expressed by other cell types in CNS?

Figure 2C - how the quantification of cell expressing IL-33 was performed? It seems that this experiment was based on the counting of positive cells, this assay is not suitable to conclude that IL-33 is being produced mainly by oligodendrocytes

*This is a fair point, and was also suggested by Reviewer 1. To further bolster our conclusion that oligodendrocytes are a key source of IL-33, we have now added additional data quantifying the loss of IL-33 positivity from cells expressing the mature myelinating oligodendrocyte marker, MBP.*

*In addition, we attempted flow cytometric approaches to quantify IL-33 expression and/or loss, but found that staining of nuclear protein in glial cells was insufficiently robust for inclusion in the manuscript. We suggest that careful quantification of intact tissue via IHC (as we have done) represents the best approach to this analysis.*

Figure 2F - how is the viral load on IL-33 conditional KO mice? Are there any differences in viral load in the CNS of these animals?

*This is a good suggestion; the suggested data have been added to the revised manuscript, appearing in Figure 2G.*

Figure 3 - Seven days after infection is probably not enough time to see cellular infiltration in the CNS, as reported by others at 9 days post infection there is a massive infiltration of CD4 and CD8 T cells in the CNS of ST2-/- mice infected with an encephalitogenic virus (DOI 10.1186/s12974-016-0628-1). Thus, to correctly conclude that the adaptive immune response is not playing a role in thei model the authors must check cellular infiltration at later time points after infection. In fact, in figure S3I and J the authors accessed the expression on CD25 and GITR at 9 days after infection, thus it seems there is a bias in the way this data is presented.

*We have now added analysis of adaptive immune cells in wild-type and IL-33 deficient mice at 14 days post infection (Fig. 3D.) These data do not reveal a significant difference between these two genotypes, a conclusion consistent with the data previously included at D7 post-infection.*

Figure S3 - these results are contradictory, previous reports from the literature shows a crucial role of CD8 T cell in controlling WNV infection, in which CD8 T cell depletion results in increased mortality and increased weight loss following WNV infection (DOI: 10.1128/JVI.78.15.8312-8321.2004). How to explain these contradictory findings?

*In the manuscript indicated by the reviewer, the authors study peripheral infection with an unattenuated strain of WNV; in this setting, CD8+ T cell responses are certainly required for viral control. Because our study focuses on responses within the CNS, we use a model of intracranial delivery of an attenuated form of WNV, a model previously used to address long-term effects of WNV infection and associated sequelae (PMID 27337340). Since this virus is delivered directly to the CNS, the requirements of T cell priming and consequent T cell responses are likely to be different. This is consistent with our findings in Fig. S3. Furthermore, we now also discuss this caveat in the discussion section of the manuscript.*

Lines 242 to 244 "We observed that IL-33 staining decreased in both the pyramidal layer and stratum lucidi of the CA3 region with increasing infectious doses of WNV-E218A (Figs 4A and 4B), consistent with IL-33 release in these regions." This experiment was based on cell staining, these findings does not support the idea of IL-33 release and could be related to cell depletion and no significant release of the cytokine. The affirmation the IL-33 is being released in this are is not entirely correct.

*Several previous studies have used loss of focal IL-33 staining as a proxy for its release (e.g. PMID 33108405.) We have edited our text to clarify the limits of this assay, but suggest that the extensive additional data presented throughout the rest of the paper using genetic systems clearly show a role for IL-33 in the response to WNV infection.*

Figure 4 - The authors claim the IL33 signaling was driving microglia activation upon WNV infection. For this experiment the authors classified activated microglia based on iba1 expression, however, since iba1 is not restricted to microglia the authors were not able to distinguish microglia from infiltrated cells, based on this is not possible to affirm that IL-33 is driving microglia activation.

*This is a good point and was also raised by Reviewer 1. As discussed in our response to Reviewer 1, we now include co-staining of Iba1 with the microglial marker TMEM119 (Fig. 4A), an analysis that confirms significant infiltration by peripheral monocytes at 10DPI. More generally, we agree that we cannot fully differentiation between microglia and engrafted peripheral macrophages. To clarify this point, we have used the term “brain macrophages” in reference to these data, and explained in the text that we cannot fully discriminate between microglia and macrophages.*

Figure 5 is not essential for the overall understanding, this figure could me moved to supplementary material to make the manuscript shorter and more straigthforward.

*We feel that Figure 5 provides important context and displays relevant data that drive subsequent interpretation, and have elected to leave it in the main body of the manuscript.*

Figure 6B is not convincing, there is only a marginal increase in positive CC-3 positive cells from IL-33-/- infected mice. In fact, all data regarding cell survival from figure 6 is not convince, there is only a minor increase in positive stained cells

*The differences are statistically significant, so we are not clear on what the reviewer intends by calling them “not convincing.” We also wish to highlight that flow cytometric analysis of WNV-infected brains also reveals an increase in staining of brain macrophages by the cell death dye Zombie NIR at multiple timepoints (Fig. 6A and S6A) as well as a loss in total number of brain myeloid cells (Fig. S6B). We therefore feel that we have convincingly shown that loss of IL-33 signaling leads to cell death within these populations.*

Lines 87 to 88

"whether IL-33 contributes to beneficial or pathogenic responses to neurotropic viral infection remains unexplored". The authors must consider some literature reports such as DOI 10.1111/imm.12988 and especially DOI 10.1186/s12974-016-0628-1 in the context of neuroinflammation and IL-33 signaling.

*These citations have been added to the manuscript.*

"Specifically, during acute infection, microglia were shown to phagocytose neuronal processes the murine hippocampus, a phenomenon that limits viral dissemination while causing disease sequelae specific to episodic spatial memory". This sentence seems odd, please review it carefully

*This sentence has been updated in the revised text to improve clarity.*

"We found that following CNS infection oligodendrocyte numbers acutely decrease, with IL-33 expression by oligodendrocytes necessary for host survival, implicating release of IL-33 from dying oligodendrocytes." Please review, I believe it refers to reduced IL-33 expression as a consequence of diminished numbers of oligodendrocyte cells

*The reviewer is correct; we have updated the text to clarify this statement.*

**Part III – Minor Issues: Editorial and Data Presentation Modifications**

**Reviewer #1**: (No Response)

**Reviewer #2**: 1) The authors nicely show a decrease in oligodendrocytes expressing IL-33, but there is also a decrease in IL-33+ astrocytes following infection (Figure 2C). This result should be mentioned in the results and discussion that astrocytes may also be a relevant source of IL-33.

*The manuscript has been revised emphasizing the possible role for astrocytes in both our results and in the discussion.*

2) ST2 is notoriously difficult to stain for using flow cytometry. The authors do not detect ST2 expression by flow in several cell types (Figures 3C and 3D), but nicely show Il1rl1 expression by real-time PCR. For example, Figure S4I nicely shows expression in microglia. Thus, examination of potential ST2-expressing population could be demonstrated by PCR, RNAscope, or other methods. Alternatively, the conclusions made from the flow cytometry staining could be discussed with the caveat that ST2 flow cytometry staining has rarely implicated cell types in the CNS that are important for responses to IL-33.

*This is a fair point; the manuscript has been edited to acknowledge the potential difficultly of detecting ST2 by flow cytometry.*

3) The authors include a description of similar studies performed with Toxo infection (line 84-86). The authors should correct the statement to reflect that astrocytes were the key responders to IL-33 in this study.

*We thank the reviewer for pointing out the need for this clarification, and have edited the manuscript accordingly.*

**Reviewer #3**: Lines 87 to 88 "whether IL-33 contributes to beneficial or pathogenic responses to neurotropic viral infection remains unexplored"
The authors must consider some literature reports such as DOI 10.1111/imm.12988 and especially DOI 10.1186/s12974-016-0628-1 in the context of neuroinflammation and IL-33 signaling.

*The manuscript has been revised to omit this statement and cite the reviewer’s provided reference.*

Lines 95 to 98 "Specifically, during acute infection, microglia were shown to phagocytose neuronal processes the murine hippocampus, a phenomenon that limits viral dissemination while causing disease sequelae specific to episodic spatial memory".

*The manuscript has been revised to clarify this statement with appropriate citations.*

Lines 103 to 106 "We found that following CNS infection oligodendrocyte numbers acutely decrease, with IL-33 expression by oligodendrocytes necessary for host survival, implicating release of IL-33 from dying oligodendrocytes."
Please review, I believe it refers to reduced IL-33 expression as a consequence of diminished numbers of oligodendrocyte cells

*The manuscript has been revised to avoid confusion and delineate these two points.*
